# An ultrasensitive NanoLuc-based luminescence system for monitoring *Plasmodium berghei* throughout its life cycle

**DOI:** 10.1186/s12936-016-1291-9

**Published:** 2016-04-21

**Authors:** Mariana De Niz, Rebecca R. Stanway, Rahel Wacker, Derya Keller, Volker T. Heussler

**Affiliations:** Institute of Cell Biology, University of Bern, 3012 Bern, Switzerland

**Keywords:** *Plasmodium berghei*, NanoLuc luciferase, Bioluminescence, Ultrabright, Ultrasensitive, PbNLuc

## Abstract

**Background:**

Bioluminescence imaging is widely used for cell-based assays and animal imaging studies, both in biomedical research and drug development. Its main advantages include its high-throughput applicability, affordability, high sensitivity, operational simplicity, and quantitative outputs. In malaria research, bioluminescence has been used for drug discovery in vivo and in vitro, exploring host-pathogen interactions, and studying multiple aspects of *Plasmodium* biology. While the number of fluorescent proteins available for imaging has undergone a great expansion over the last two decades, enabling simultaneous visualization of multiple molecular and cellular events, expansion of available luciferases has lagged behind. The most widely used bioluminescent probe in malaria research is the *Photinus pyralis* firefly luciferase, followed by the more recently introduced Click-beetle and Renilla luciferases. Ultra-sensitive imaging of *Plasmodium* at low parasite densities has not been previously achieved. With the purpose of overcoming these challenges, a *Plasmodium berghei* line expressing the novel ultra-bright luciferase enzyme NanoLuc, called PbNLuc has been generated, and is presented in this work.

**Results:**

NanoLuc shows at least 150 times brighter signal than firefly luciferase in vitro, allowing single parasite detection in mosquito, liver, and sexual and asexual blood stages. As a proof-of-concept, the PbNLuc parasites were used to image parasite development in the mosquito, liver and blood stages of infection, and to specifically explore parasite liver stage egress, and pre-patency period in vivo.

**Conclusions:**

PbNLuc is a suitable parasite line for sensitive imaging of the entire *Plasmodium* life cycle. Its sensitivity makes it a promising line to be used as a reference for drug candidate testing, as well as the characterization of mutant parasites to explore the function of parasite proteins, host-parasite interactions, and the better understanding of *Plasmodium* biology. Since the substrate requirements of NanoLuc are different from those of firefly luciferase, dual bioluminescence imaging for the simultaneous characterization of two lines, or two separate biological processes, is possible, as demonstrated in this work.

## Background

Malaria is a life-threatening disease caused by *Plasmodium* parasites, which are transmitted to humans through the bite of infected *Anopheles* mosquitoes. The five *Plasmodium* species able to infect humans are *Plasmodium falciparum, Plasmodium vivax, Plasmodium malariae, Plasmodium ovale* and *Plasmodium knowlesi*, all of which vary in their clinical manifestations and severity. Currently, there are 200 million clinical cases and over 500,000 deaths reported per year, most of which occur in sub-Saharan Africa [1]. Major challenges presently existing for malaria elimination programs include the emergence of parasites showing multidrug-resistance to current anti-malarials, increasing development of resistance by mosquitoes to insecticides, and the presence and widespread distribution of parasite species such as *P. vivax*, that produce dormant stages in the liver and are thus capable of inducing multiple relapses [2]. The malaria eradication agenda envisages advances in vaccine development against pre-erythrocytic stages, anti-malarial compound development, vector control, and sensitive diagnostic and surveillance tools in endemic areas [3]. In addition, there is a pressing need for improving the general understanding of parasite biology, and the identification of targets for generation of genetically attenuated parasites (GAPs) and anti-malarial activity. Animal models of malaria, and particularly rodent models, have proven to be extremely valuable tools for basic research on parasite and host biology, host-pathogen interactions, and anti-malarial drug activity [4–6].

Bioluminescence is found in nature in a large diversity of organisms. The generation of light occurs when a luminogenic substrate is oxidized, resulting in the emission of photons that can be accurately measured by sensitive specialized equipment such as luminometers or CCD cameras. *Plasmodium falciparum* and several rodent *Plasmodium* strains have already been engineered to express bioluminescent reporters. The uses of bioluminescence in *Plasmodium* were recently reviewed by Siciliano and Alano [7]. Among the applications of bioluminescence in *Plasmodium* research, some of the most relevant studies include the *in vivo* and in vitro assessment of parasite growth in liver [8–15] and blood stages [14]; schizont sequestration in vivo ([16–19], De Niz et al., unpublished); analysing stage-specificity of promoters and other regulatory elements [20–33]; testing anti-malarial activity of drugs targeting liver [11, 34–40], and blood stage parasites [41–46]; screening for transmission-reducing activity of anti-malarials [47–50]; functional studies of parasite proteins and/or the adequacy of parasite attenuation by genetic manipulation ([16, 18, 51, 52]; De Niz et al., unpublished); evaluation of anti-malarial immunity [53–55]; visualization of sporozoites in the skin [56], and specific differentiation of gametocyte stages (I-V) [47].

**Fig. 1:**
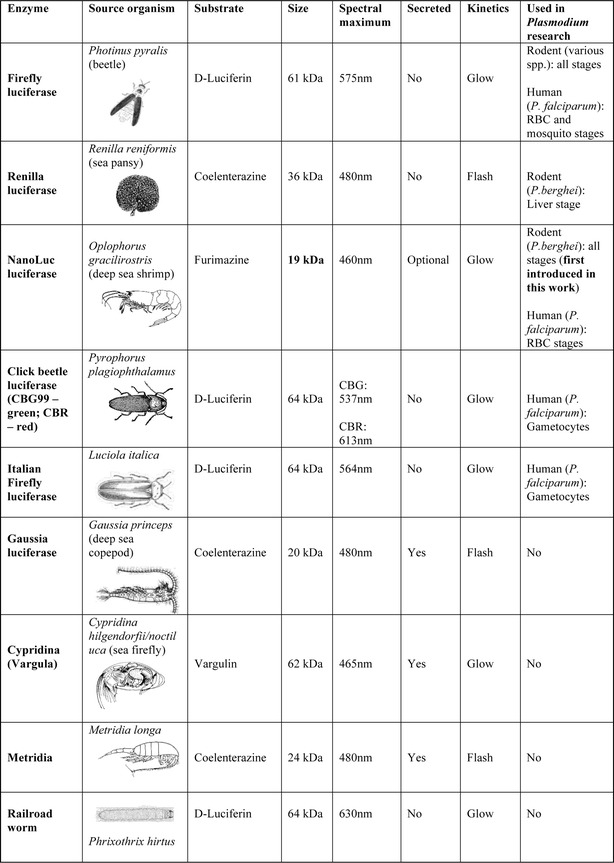
Luminescent proteins used in biomedical research

The most widely used luciferase in *Plasmodium* research has by far been firefly luciferase, isolated from the firefly *Photinus pyralis* [57, 58]. Other luciferases also previously used in malaria include Renilla luciferase, isolated from the sea pansy *Renilla reniformis* [59, 60], and Click beetle luciferases isolated from the beetle *Pyrophorus plagiophthalamus* [61, 62]. A wide variety of living organisms, including arthropods, annelids, molluscs, fish, cnidarians, protists, as well as various microorganisms, are able to produce light or ‘bioluminesce’. Bioluminescent proteins from many of these organisms have been isolated for use in biomedical research. Figure 1 shows the various luciferases currently available, with their key luminescence properties (Diagram references [63–69]). Despite the wide variety of organisms capable of producing luminescent proteins, bioluminescence imaging in biomedical research is thought to be relatively limited for simultaneous measurements. This is in contrast to the wide variety of proteins available for fluorescence imaging. In addition to the luciferases listed in Fig. 1, novel artificial methods to shift the emission spectra of currently existing luciferases and the generation of artificial luciferases and substrates, will continue to expand the field of luminescence imaging and its application to various areas of research, including malaria.

While firefly luciferase has provided enormous advantages for assay scalability in vivo and in vitro and has multiple applications in biomedical research, recently, a novel 19 kDa enzyme named NanoLuc, was synthetically generated following purification of the natural enzyme from the deep sea shrimp *Oplophorus gracilorostris*. NanoLuc possesses 150-fold brighter luminescent signal than firefly or Renilla [70]. Aside from its high level of luminescence, NanoLuc is able to sustain signal intensity with a relatively long half-life of 2 h, and generates low background signal making it an efficient, highly stable, and highly sensitive assay. In addition to optimal activity of the enzyme, suitable substrates were investigated to further maximize protein stability and light output. The coelenterazine analogue furimazine, in combination with NanoLuc, was found to produce the brightest output, and enhanced stability. Despite its relatively recent introduction, NanoLuc has already been used to generate a wide variety of transgenic organisms and cells for in vivo [71–78] and in vitro [79–96] studies, most of which have reported the considerable advantages of NanoLuc as a luminescent system, including the possibility of doing dual or multiplex assays in combination with Gaussia [82], or firefly [72, 94] luciferases. Among the transgenic organisms using this technology, NanoLuc-expressing *P. falciparum* parasites (termed PfNLuc) have already been generated, and used for inhibitory compound screening assays, and protein secretion studies [97].

In this work, NanoLuc is introduced as a reporter in *Plasmodium berghei*. PbNLuc parasites were generated to constitutively express NanoLuc under the ef1α promoter. All stages of the *P. berghei* life cycle were assessed in terms of minimum detection limit of the assay at low parasite densities, kinetics of parasite development, and sensitive detection of *P. berghei* egress in its transition from the liver to the blood stages. Also as proof of concept, the assay is used in a dual format together with firefly-luciferase to study co-infections in vivo.

## Methods

### Ethics statement

Balb/c and C57BL/6 studies were carried out under the approval of the Animal Research Ethics Committee of the Canton Bern, Switzerland (Permit Number: 91/11 and 81/11); the University of Bern Animal Care and Use Committee, Switzerland; and the German Tierschutzgesetz (Animal Rights Laws). For all studies, females 5–8 weeks of age, weighing 20–30 g at the time of infection were used. Mice were purchased from Harlan or Charles River laboratories. All measurements involving in vivo bioluminescence were performed under isofluorane anaesthesia. Blood feeding was performed under ketavet/dorbene anaesthesia, and all efforts were made to minimize suffering.

### Plasmid construction

The pL0017 vector (obtained through the Malaria Research and Reference Reagent Resource Center [(MR4; http://www.mr4.org/) (MR4 no., MRA-786)] allows constitutive expression of GFP in *P. berghei* parasites, under the control of the ef1α promoter, either by episomal expression, or by integration of the plasmid sequence into the *cssu/dssu* (c-type small subunit/d-type small subunit) ribosomal gene locus of the parasite. The NLuc sequence was amplified from the pNL1.1 (NLuc) vector (Promega) using Phusion ^®^ Taq High-Fidelity DNA polymerase, and the primers 5′-GGATCCATGGTCTTCACACTCGAAGATTTC-3′ and 5′-TCTAGATTACGCCAGAATGCGTTGCG-3′. The pL0017 plasmid was cut at *Bam*HI and *Xba*I sites, and the GFP sequence excised. NLuc was introduced into the pJET vector as an intermediate step, and then introduced into the pL0017 vector using the *Bam*HI and *Xba*I restriction sites to create plasmid pL0017-NLuc. Plasmid DNA was linearized with *Apa*I and *Sac*II, and 5–10 μg of linearized plasmid DNA was used for transfection of either schizonts or detached cells.

### Schizont transfection

Six to eight-week old Balb/c mice were infected with PbmCherry_Hsp70_ [98] until the parasitaemia reached 5–6 %. Mice were bled by intracardiac puncture and schizont cultures generated. Schizonts were then isolated as previously published [99]. Parasites were transfected with pL0017-NLuc, and the transfectant injected into one Balb/c mouse by intravenous injection. One day post-infection, the mouse was treated with 70 mg/ml pyrimethamine in drinking water for positive selection of PbmCherry_Hsp70_NLuc_ef1α_.

### Detached cell transfection

An alternative method tested for the first time, was transfection of liver stage detached cells. HepG2 cells were seeded in a 24-well plate (Greiner BioOne) and the next day infected with PbmCherry_Hsp70_ sporozoites. Infected HepG2 cells were maintained as described in further sections. Sixty-five hours after infection and upon completion of the liver stage of infection, detached cells or merosomes were collected from the supernatant and centrifuged for 10 s at 1600 rpm. Merosomes and detached cells were then transfected as previously published [99], with 5–10 µg of linearized pL0017-NLuc and injected into the tail vein of a naïve Balb/c mouse. One day post-infection, the mouse was treated with 70 mg/ml pyrimethamine in drinking water for positive selection of PbmCherry_Hsp70_NLuc_ef1α_.

### Clone selection

For clone selection, *Anopheles stephensi* mosquito feeds were performed as described below, and at day 18 post-feed, mosquito salivary glands were dissected for infection of HepG2 cells (as described below). Upon completion of the liver stages, six individual detached cells were isolated using previously published methodology [100], and injected into separate mice. 10,000 infected red blood cells (iRBCs) were isolated from each mouse, and probed using the NanoGlo™ (Promega) assay as described below. The clones consistently displaying the brightest signal were selected for downstream analyses.

### Luciferase assay

Transgenic parasites from all stages were lysed in 20 μl of 1 × Passive Lysis Buffer (1 × PLB) (Promega) as recommended by the manufacturer. For firefly luciferase measurements, these 20 μl lysates were mixed with 100 μl of Luciferase assay reagent, containing the luciferase assay substrate luciferin dissolved in Luciferase Assay Buffer (LAB), and measured within 30 s in an IVIS Lumina II equipment, with 45 s exposure time, and gain adjusted to maximum. For NanoLuc assays, lysates were mixed with 50 μl of the NanoGlo™ luciferase assay reagent. The NanoGlo™ luciferase assay reagent was optimized for maximum signal by adding one volume of NanoGlo™ luciferase assay substrate to 50 volumes of NanoGlo™ Luciferase assay buffer, as recommended by the manufacturer. Optimization for imaging parasites at low concentrations included both, finding the minimum concentration of parasites detectable in the 1:50 assay mixture, and the maximum dilution of NanoGlo™ luciferase assay substrate that would still enable optimal signal detection while allowing for significant cost reductions and maximum use. Concentrations tested other than 1:50, included 1:100, 1:200, 1:500 and 1:1000, all of which allowed detection of parasites at low, medium and high concentrations. Single parasites of all *Plasmodium* stages were only detected using maximum detection settings (45 s exposure, and 1:50 NanoLuc concentration).

### Parasite maintenance in mosquitoes

Balb/c mice were treated with phenylhydrazine three days prior to i.p infection with *P. berghei* expressing mCherry and either Firefly or NanoLuc luciferases (PbmCherry_Hsp70_FLuc_ef1α_ or PbmCherry_Hsp70_NLuc_ef1α_). After 3 days of infection, exflagellation was assessed. The infected mice were then each used to feed 100–150 *Anopheles stephensi* female mosquitoes. Mice were anaesthetized with a combination of Ketasol/Dorbene anaesthesia, and euthanized with CO_2_ after completion of the feed. Afterwards, mosquitoes were fed until use with 8 % fructose containing 0.2 % PABA.

### In vitro luciferase assays for oocyst measurements

Seven to nine days following the blood meal on infected mice, 100 mosquitoes from three different cages infected either with PbmCherry_Hsp70_NLuc_ef1α_ (PbNLuc) or PbmCherry_Hsp70_FLuc_ef1α_ (PbFLuc) were sorted using an epifluorescence binocular microscope (Olympus SZX10 using a U-HGLGPS UV lamp), and mCherry-positive mosquitoes selected, dissected and the midguts isolated to evaluate oocyst numbers and luciferase expression. For correlation between fluorescence and luminescence assays, 100 dissected midguts were transferred in 1 × PBS onto a coverslip, and imaged immediately using a 20× objective, of an upright epifluorescence microscope (Leica DM 5500 B). Four z-stacks from each midgut were obtained to allow for quantification of oocytsts over the entire midgut area. Upon finalizing image acquisition, midguts were immediately transferred to the 20 μl of 1 × PLB for downstream analysis. For prevalence assays, 200 mosquitoes were not sorted prior to dissection or homogenization, but randomly selected from 3 independent feed cages harbouring no more than 150 mosquitoes each. Bioluminescent measurements of all samples were performed in 96-well, flat-bottom, non-binding, black plates (Greiner BioOne). For consistent and unsaturated oocyst measurements, the optimal NanoGlo™ luciferase assay substrate dilution used for probing the midguts was 1:1000. The midguts of uninfected mosquitoes were used as negative controls.

### In vitro luciferase assays for sporozoite measurements and limit of detection

Sixteen to 28 days post-feed, mosquitoes infected with PbmCherry_Hsp70_NLuc_ef1α_ (PbNLuc) or PbmCherry_Hsp70_FLuc_ef1α_ (PbFLuc) were dissected for extraction of their salivary glands. Sporozoites were counted using a Neubauer chamber, and serial dilutions of sporozoites made in 1 × PBS. The final diluted samples were centrifuged and the pellet (10 μl) transferred to 20 μl of 1 × PLB for downstream analysis. For imaging, a range between 1 and 100,000 sporozoites, different dilution sets from 20 separate mosquitoes were divided into independent 96-well plates to enable different imaging settings for maximum signal detection of samples at low densities (i.e. 1–100 sporozoites), while avoiding pixel saturation of samples at high densities (i.e., 10,000–100,000 sporozoites). For consistent and unsaturated sporozoite measurements, the optimal NanoGlo™ luciferase assay substrate dilution used was 1:250. Salivary glands of uninfected mosquitoes were used as negative controls.

### In vitro luciferase assays for liver stage measurements

Human hepatoma cells (HepG2 line) (European Collection of Cell Culture) were maintained in complete Minimum Essential Medium (cMEM) containing Earle’s Salts, supplemented with 10 % heat-inactivated fetal calf serum (FCS), 2 mM l-Glutamine, and 100 U Penicillin, 100 μg/ml Streptomycin (all from PAA laboratories, E15-024, A15-101, P11-010, M11-004). Cells were kept at 37 °C in a 5 % CO_2_ cell incubator and were split every 4 days by treatment with accutase. For in vitro bioluminescence assays, 30,000 cells were seeded into wells of 24-well plates, and infected 18-24 h afterwards with 10,000 PbmCherry_Hsp70_NLuc_ef1α_ (PbNLuc) or PbmCherry_Hsp70_FLuc_ef1α_ (PbFLuc) sporozoites. Upon infection, sporozoites and HepG2 cells were incubated together in cMEM + 2.5 μg/ml amphothericin (AT-MEM) to avoid fungal contamination. At 6, 12, 24, 36, 48, and 56 h post-infection, total parasite numbers were quantified by fluorescence, and HepG2 cells detached using 100 μl of accutase. Cells were quantified using a Neubauer chamber, and specific parasite numbers isolated (ranging from 1 to 1000). The diluted samples containing the desired number of parasites were lysed in 20 μl of 1×PLB for downstream analysis. For isolation of single later stage parasites (36 h onwards), the cells detached by accutase treatment were imaged using an epifluorescence microscope to ensure capture of a single parasite (in a method analogous to that described by Stanway et al. [100]. Lysates were transferred to 96-well black plates, and imaged immediately at every time point using an IVIS Lumina II. For consistent and unsaturated measurements, the optimal NanoGlo™ luciferase assay substrate dilution used for probing liver stage parasites was 1:250. Uninfected HepG2 cells were used as negative controls.

### In vitro luciferase assays for liver stage egress, detached cell formation and merozoite numbers

In order to investigate detached cell formation by PbmCherry_Hsp70_NLuc_ef1α_ (PbNLuc) parasites, HepG2 cells were infected as previously described, and at 63–65 h post-infection, the supernatant of the 24 well-plates, where detached cells (representing successfully completed liver stage development) are located, was collected for downstream analysis. For collection of single detached cells, the method previously published by Stanway et al. [100] was used. Prior to collection and lysis, individual detached cells were imaged by fluorescence using an inverted fluorescence microscope (Leica DMI 6000B with an incubation chamber), and a series of 3–4 z-stacks obtained for measurements. Upon collection, individual detached cells were lysed in 1xPLB, and assayed by luminescence. For consistent and unsaturated measurements, the optimal NanoGlo™ luciferase assay substrate dilution used for probing detached cells was determined to be 1:250. In order to determine the number of merozoites per individual detached cell, a previously published method [101] was used. Namely, from the fluorescence images, the radius of the detached cell was determined, and the volume of the detached cell (*V*_*DC*_) was calculated by the formula $V_{DC} = \frac{4}{3}\pi r^{3}$. Sturm et al. previously determined the volume of individual merozoites (*V*_*m*_) to be equal to 2.66 μm^3^ [101]. An approximation of the number of merozoites (*N*_*m*_) per detached cell is, therefore, calculated as $N_{m} = \frac{{V_{DC} }}{{V_{m} }}$.

### *In vivo* luciferase assays for liver stage egress and pre-patency determination

To determine the pre-patency period detectable by NanoLuc, a total of 20 mice were infected with 5000 sporozoites from PbmCherry_Hsp70_NLuc_ef1α_-infected mosquitoes by tail-vein injection. 2 µl of blood were taken by tail puncture at 12 and 24 h post-infection and from 36 h onwards, 4-hourly measurements were made until 70 h post-infection. This blood was lysed in 20 µl of 1 × PLB, and for maximum sensitivity, probed with 1:50 NanoGlo™ luciferase assay substrate dilutions. To determine in vitro sensitivity, individual detached cells from infected HepG2 cultures (described above) were isolated as previously described [100], and imaged using an epifluorescence microscope for downstream volume calculations. Following imaging, each individual detached cell was transferred to 20 µl of 1 × PLB. Images of blood and detached cells were acquired immediately using an IVIS Lumina II equipment. For maximum sensitivity for appearance of parasites in the blood, a 1:50 dilution of NanoGlo™ luciferase assay substrate was used.

### In vitro luciferase assays for blood stage measurements

For blood stage measurement curves and assays for detecting the minimum sensitivity limit, parasites were diluted to ranges between 1 and 100,000. Each of the diluted samples were lysed in 1 × PLB, and imaged immediately. For calculation of the parasitaemia, three separate groups of five mice each (Balb/c and C57BL/6) were infected with 10^6^ schizonts of either PbmCherry_Hsp70_NLuc_ef1α_, or PbmCherry_Hsp70_FLuc_ef1α_. 2 µl of blood were obtained daily by tail vein prick, lysed in 20 µl of 1 × PLB, and probed immediately. Every day, thin smears were made on glass coverslips, and Giemsa stains performed for microscopic quantification of parasitaemia to assess correlations between luminescence values and parasitaemia in blood smears. For analysis of sequestration, mice were injected with either 10^6^ PbmCherry_Hsp70_NLuc_ef1α_ schizonts, or with a mixture of PbmCherry_Hsp70_NLuc_ef1α_ and PbmCherry_Hsp70_FLuc_ef1α_ (5 × 10^5^ schizonts of each line), and a synchronous infection allowed to establish. 2 µl of blood were extracted bi-hourly within the first 28 h post-schizont injection via tail vein puncture, lysed in 20 µl of 1 × PLB, and imaged. For maximum sensitivity of single blood stages, a 1:50 dilution of NanoGlo™ luciferase assay substrate was used. In all other assays for in vivo parasitaemia measurements, a 1:250 dilution was used. Erythrocytes from uninfected mice were used as negative controls.

### Statistical analyses

All data analysis of luminescence values was performed using the LivingImage 4.1 software. Analyses for Pearson correlation coefficients, Student’s *t* tests, and linear regressions were carried out using STATA 13.0 software. Other than the STATA-generated graphics, all other graphics were generated using the Prism 6.0 software. All parasite measurements including diameter, radius, and area, were performed using plugins freely available in Fiji. Significance in all assays was determined upon *p* values being equal to, or less than 0.05.

## Results

### PbNLuc parasite generation and clone selection

For the generation of the pNLuc plasmid, the GFP coding sequence from the pL0017 plasmid was excised, and replaced by the NanoLuc coding sequence, thereby allowing expression of NanoLuc under the control of the ef1α promoter. PbmCherry_Hsp70_ parasites [98] were transfected with the pNLuc construct, and this resulted in single cross-over integration events. Individual Pb mCherry_Hsp70_NLuc_ef1α_ clones were isolated by detached cell injection (89) into six mice. A drop of blood was isolated from all mice harbouring synchronous ring infections. Parasites were probed with NanoGlo™ according to manufacturer’s instructions, and luminescence measured with an IVIS Lumina II imager. Measurements were repeated three times at different days post-infection, isolating equal numbers of iRBCs by dilution. Although signal intensity between most clones did not differ significantly, the clone that consistently showed the highest signal was chosen for downstream analyses.

### Bioluminescence-based analysis of PbNLuc oocysts on the mosquito midgut

**Fig. 2:**
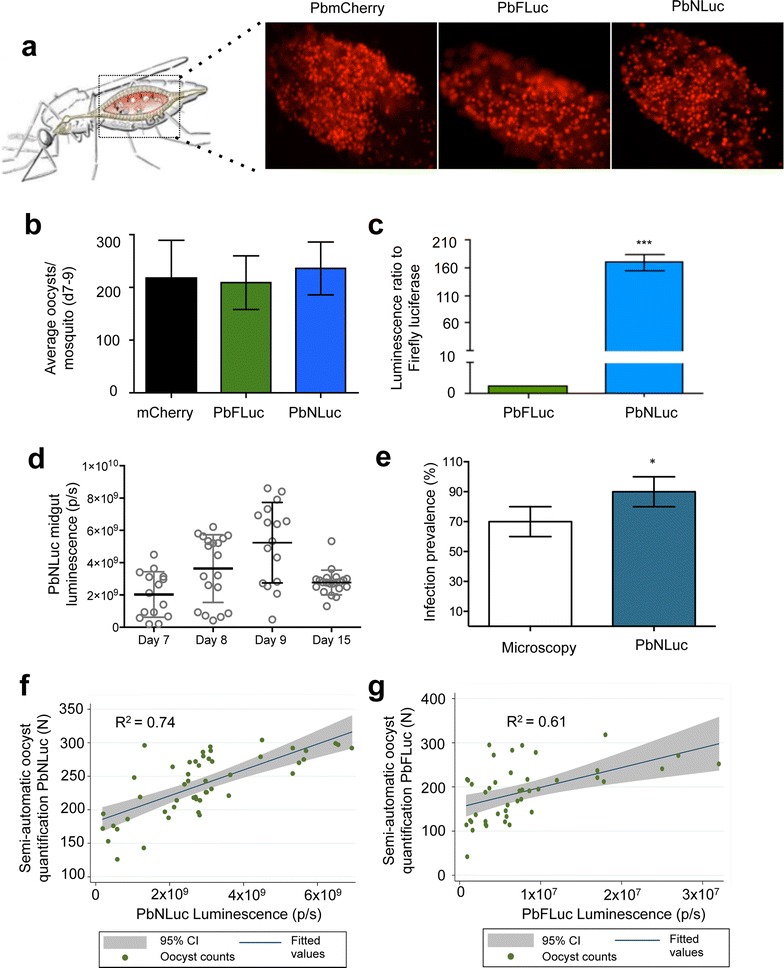
Bioluminescence imaging of oocysts in mosquito midguts. **a** Representative fluorescence images of dissected midguts from PbmCherry, PbFLuc, and PbNLuc-infected mosquitoes. **b** Average oocyst numbers in PbmCherry, PbFLuc and PbNLuc-infected midguts at days 7–9 post-feed. **c** Luminescence ratio obtained from 50 mosquitoes infected with PbFLuc or PbNLuc lines (PbFLuc set as 1). **d** Comparative analysis of prevalence of PbNLuc infection in mosquitoes. Luminescence values of PbNLuc-infected mosquitoes at days 7–9 post-feed, prior to oocyst rupture and sporozoite egress, and at day 15 post-feed, following initial sporozoite egress events. **e** At day 9 post-feed, 200 mosquitoes were sorted by fluorescence into ‘infected’ and ‘uninfected’. Based on these data, the prevalence of infection was calculated. The same mosquitoes were dissected and the midguts lysed for bioluminescence assays and infection prevalence calculation. **f** Scatter plot showing correlation and 95 % confidence interval range (CI) of PbNLuc bioluminescence values to fluorescence-based semi-automated counts. 100 individual PbNLuc-infected mosquitoes (represented by each *dot*) were dissected, and their midguts extracted. Oocyst numbers for each individual midgut were quantified using a semi-automated fluorescence-based macro (*y-axis*). Midguts were then lysed and imaged by luminescence (*x-axis*). **g** Scatter plot showing correlation and 95 % CI range of PbFLuc bioluminescence values to fluorescence-based semi-automated counts. Experimental setup as described for (**f**). [*Graphs* are the result of triplicate experiments. Luminescence values are expressed as photons per second (p/s); *p < 0.05; ***p < 0.001; *error bars* in graphs represent standard deviations (SD)]

Microscopy-based imaging and quantification of PbmCherry_Hsp70_, PbmCherry_Hsp70_FLuc_ef1α_ [39] (henceforth referred to as PbFluc) and PbmCherry_Hsp70_NLuc_ef1α_ (henceforth referred to as PbNLuc) in mosquito midguts showed that all three lines strongly express mCherry (Fig. 2a) (Diagram adapted from [102]) and have equivalent average numbers of oocysts per mosquito on days 7–9 post-feed (Fig. 2b). For comparison of PbNLuc and PbFLuc luminescence intensities, ten pools of five midguts with approximately equal numbers of oocysts from PbFLuc- and PbNLuc-infected mosquitoes were dissected, lysed, and their bioluminescence measured. Midguts infected with PbNLuc oocysts showed 160- to 180-fold higher signal (*p* < 0.05) than midguts infected with PbFLuc oocysts (Fig. 2c).

In order to determine the NanoLuc assay’s sensitivity in detecting changes in average numbers and sizes of oocysts throughout their development on midguts, 50 midguts from mosquitoes in three independent cages were dissected and measured at days 7–9 and 15 post-feed. Although detecting small variations (in the range of tens of oocysts) is challenging using firefly luciferase, NanoLuc-based luminescence in PbNLuc parasites allowed detection of a clear progressive increase in intensity from days 7–9 post-feed, which decreased again at day 15 coinciding with sporozoite egress and migration to the salivary glands (Fig. 2d).

As proof of principle for the use of PbNLuc in assessment of infection prevalence in mosquitoes, a total of 200 mosquitoes were sorted by fluorescence into two groups consisting either of mCherry-positive (infected), or mCherry-negative (non-infected) mosquitoes. By microscopy alone, the prevalence of infection was calculated to be 70 % (SD, 10.5 %). These mosquitoes were then dissected and their midguts imaged by bioluminescence. The prevalence estimate detected by luminescence of this same group, was 88 % (SD, 12 %) (Fig. 2e), emphasizing the enhanced sensitivity of the assay for determining presence or absence of midgut oocysts by NanoLuc luminescence rather than fluorescence. In addition, the assay avoids observer bias in terms of assessment of fluorescence at low oocyst densities. While fluorescence-based sorting is to an extent subjective, sensitive bioluminescence imaging enables non-subjective, quantitative assessment of infection prevalence.

Having proven the enhanced sensitivity of PbNLuc over fluorescence for assessment of prevalence, a next step was to explore the correlation of PbNLuc luminescence with oocyst numbers in individual midguts. At days 7–9 post-feed, 100 infected midguts of PbFLuc or PbNLuc-infected mosquitoes were dissected, imaged by fluorescence for manual or semi-automated quantification of oocysts, and then immediately lysed for imaging by luminescence. Oocyst numbers were quantified by two different methods, namely, a previously published semi-quantitative method based on fluorescence [103]; and light emission values based on NanoLuc or firefly luciferase bioluminescence. Figure 2f, g show fitted correlations and 95 % confidence intervals of bioluminescence versus automated fluorescence counts (each dot corresponds to the measurement of one individual midgut). PbNLuc shows a Pearson correlation coefficient of 0.74 (Fig. 2f), while PbFLuc shows a correlation coefficient of 0.61 (Fig. 2g). This suggests that the increased brightness of NanoLuc luciferase in PbNLuc parasites results in more sensitive measurements, allowing differences of even ten to a hundred oocysts to be distinguished. Nevertheless, a perfect correlation between counts and luminescence is not expected, as individual oocysts may greatly vary in their sporozoite load. This phenomenon has previously been reported in luminescence-based assays for transmission assessment [50].

### Bioluminescence-based analysis of PbNLuc sporozoites and limit of detection in vitro

**Fig. 3:**
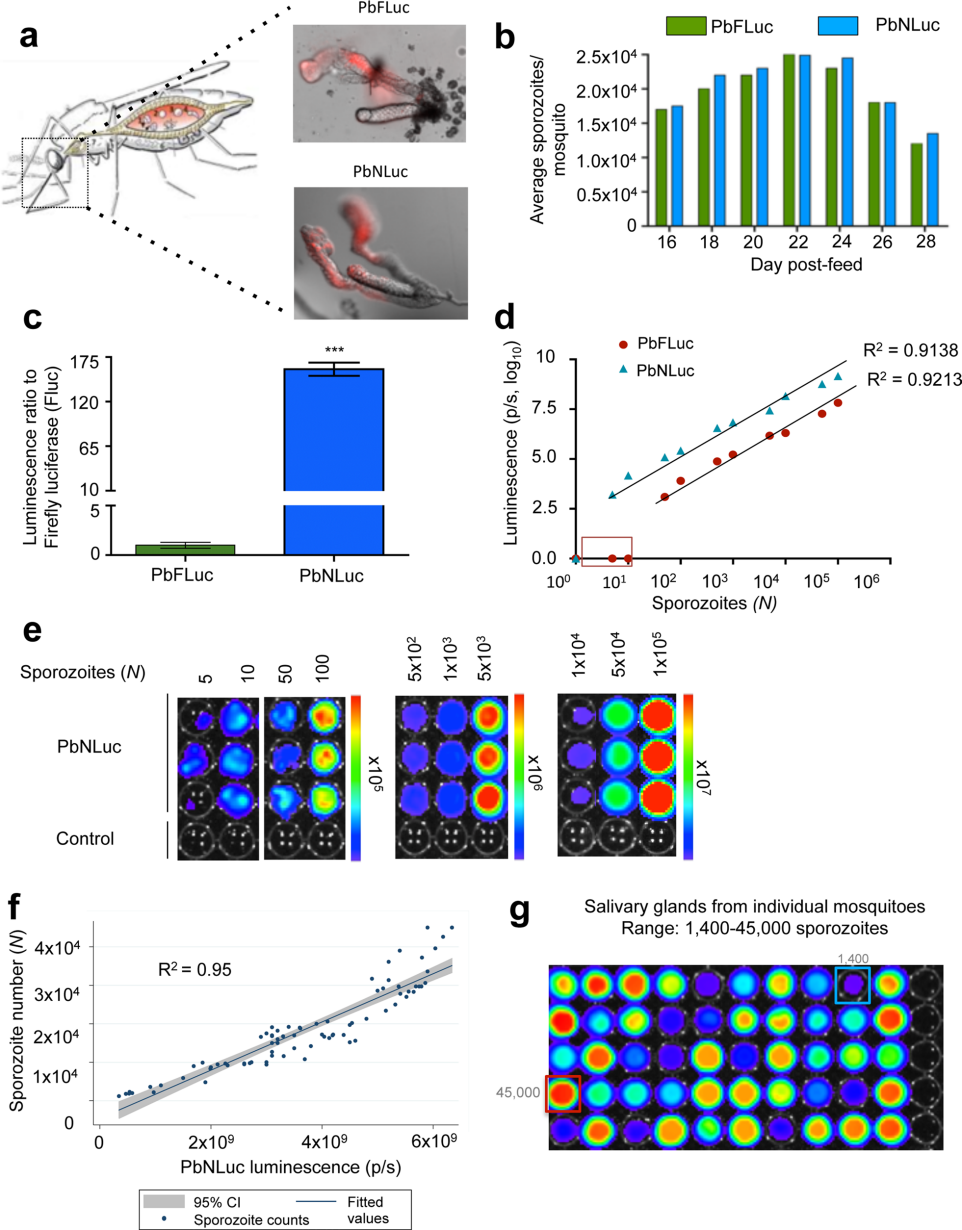
Bioluminescence imaging of sporozoites in mosquito salivary glands. **a** Representative images obtained by fluorescence microscopy, of dissected salivary glands from PbFLuc, and PbNLuc-infected mosquitoes. **b** Average sporozoite numbers per mosquito at days 16–28 post-feed, in groups of PbFLuc and PbNLuc-infected mosquitoes. **c** Luminescence ratio of PbFLuc and PbNLuc sporozoites. Four sets of 50,000 sporozoites from 10 separate mosquitoes each, for either PbFLuc or PbNLuc, were isolated from the salivary glands, and their luminescence acquired. The value for PbFLuc luminescence was set to a value of 1, and the value of PbNLuc luminescence expressed in relation to the PbFLuc value. **d** Correlation of luminescence values of PbNLuc and PbFLuc, and detection limit obtained by imaging 1–10^6^ sporozoites (Square at axis shows no signal from PbFLuc at low sporozoite densities). 20 independent mosquitoes were dissected, and their sporozoites diluted to the specific values shown in the figure. R^2^ corresponds to values over the detection limit of each respective parasite line. **e** Images from 3 independent dilutions of sporozoites (obtained from pools of 20 independent mosquitoes each). Measurements of different sporozoite concentrations were made in separate plates to avoid under-detection, or signal saturation in the presence of extremely high luminescence values. **f** Scatter plot showing correlation and 95 % CI range of PbNLuc luminescence values of 100 individual mosquitoes to manual counts in a Neubauer chamber. 100 individual PbNLuc-infected mosquitoes (represented by each *dot*) were dissected, and their salivary glands extracted. Sporozoite numbers for each individual mosquito dissected were quantified manually using a Neubauer chamber (*y-axis*). Sporozoites were then lysed and imaged by luminescence (*x-axis*). **g** Representative IVIS image of individual dissected and disrupted salivary glands (salivary glands from one uninfected mosquito is used as control in every row) Blue and red outlines show, respectively, minimum and maximum detected sporozoite loads. (luminescence values are expressed as photons per second (p/s) (linear or logarithmic) [***p < 0.001; *error bars* in all graphs represent standard deviations (SD)]

In the *Plasmodium* life cycle, a critical step following oocyst formation is the production of sporozoites, as these latter forms ultimately enable mosquito-to-human transmission. Having explored the suitability and sensitivity of PbNLuc to assess oocyst formation and development, this section explores the sensitivity and limit of detection of PbNLuc to quantify sporozoites. Fluorescence-based images of sporozoites within salivary glands of mosquitoes infected with PbFLuc or PbNLuc were obtained (Fig. 3a) (Diagram adapted from [102]), and the number of sporozoites in both luminescent lines were quantified at days 16, 18, 20, 22, 24, 26 and 28 days post-feed. PbFLuc and PbNLuc show equivalent sporozoite counts at all days post-feed, with maximum sporozoite loads detected between days 20 and 24 post-feed (Fig. 3b) suggesting no differences in egress and development between the two parasite lines. Next, equal numbers of PbFLuc and PbNLuc sporozoites (four separate pools of 50,000) were lysed, and their luminescence measured for comparison of their luminescence intensities. Luminescence values of PbNLuc sporozoites were 170-fold higher than luminescence values for equal numbers of PbFLuc sporozoites (Fig. 3c), confirming the results presented in Fig. 2c. To determine the detection limit of NanoLuc for sporozoites, serial dilutions of PbNLuc sporozoites were generated in triplicate, and lysed for measurement. While the minimum number of detectable PbFLuc sporozoites was between the range of 50 and 100, single PbNLuc sporozoites were detectable (subject to a sporozoite actually being present in the well) (Fig. 3d, e). The reason why 5 parasites per well are presented was the experimental setup itself: using serial dilutions, the probability of one single sporozoite being present in each well was 10 %. Nevertheless, every time there was a single sporozoite per well, signal was detected. Sporozoite numbers showed high correlation values with the log values of luminescence (R_2_ = 0.913 for PbNLuc, and R_2_ = 0.921 for PbFLuc) (Fig. 3d). This highly significant correlation shows the success of the assay in terms of both detection of very low sporozoite densities, and detection of small variations in sporozoite numbers. The detection limit of PbNLuc, however, is higher than that of PbFLuc. As a proof of principle for its application, salivary glands of 100 PbNLuc-infected mosquitoes were dissected, and the correlation between sporozoite numbers (as assessed by manual counting) and luminescence was calculated. Each dot represents the sporozoites of a single mosquito, as assessed by luminescence (x-axis) and manual counts (y-axis). On one hand, the test confirmed a high correlation between sporozoite numbers and luminescence values (R_2_ = 0.95) when applied to single mosquitoes (Fig. 3f). On the other hand, the assay also shows that given this high correlation, visual inspection of plates to estimate variability in the overall burden of sporozoites per mosquito is possible (Fig. 3g).

### Bioluminescence-based analysis of PbNLuc liver stages and limit of detection in vitro

**Fig. 4:**
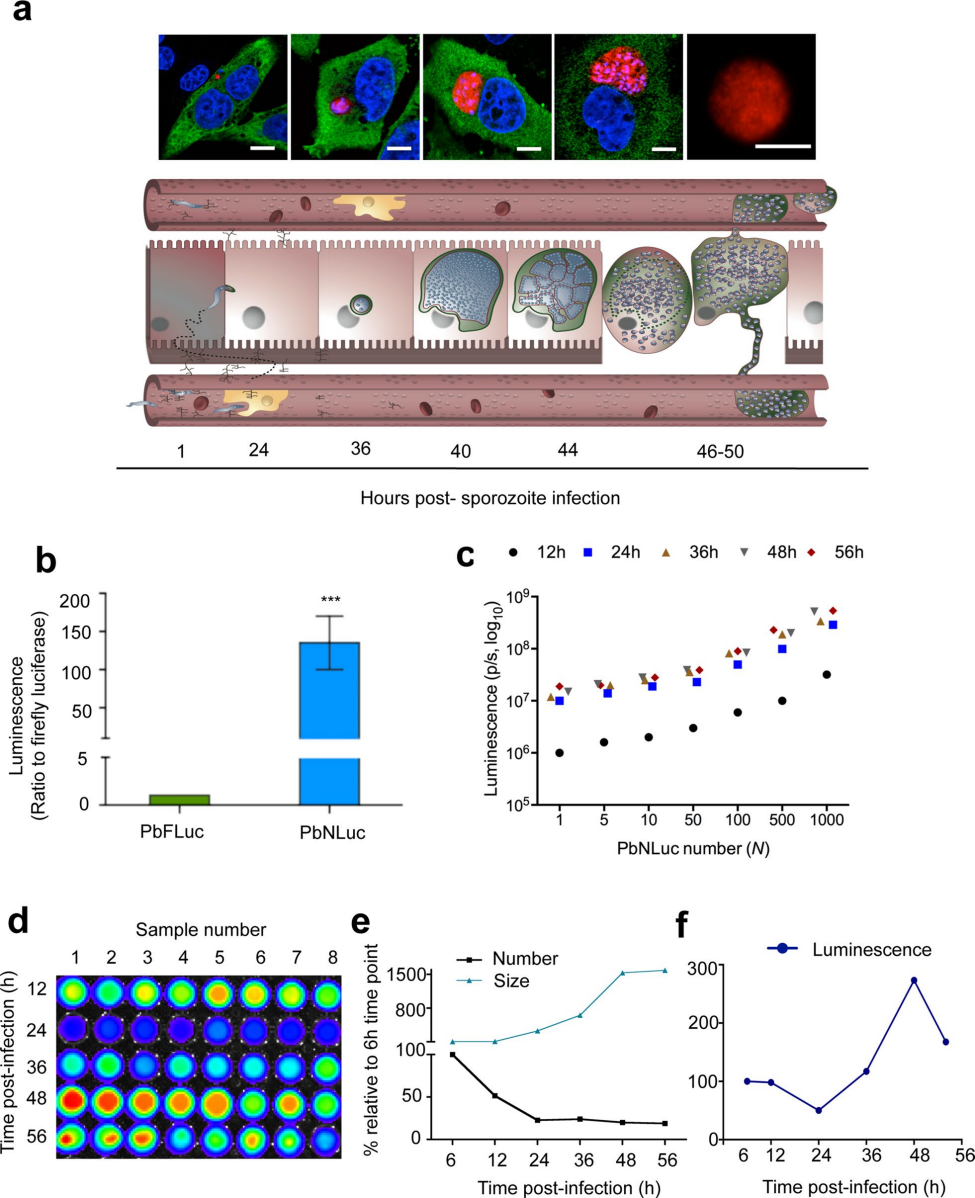
Bioluminescence imaging of *P. berghei* pre-erythrocytic stages. **a** Fluoresence images of liver cells infected with PbNLuc, showing parasite development from 2 h to egress. Scale bar is equal to 15 μm. The lower panel shows a schematic representation of liver stage development at the corresponding time points. **b** Ratio of luminescence between PbFLuc and PbNLuc signal arising from 6 independent sets of HepG2 cells infected with 10,000 sporozoites each, and lysed at 40 h post-infection. The value for PbFLuc luminescence was set to a value of 1, and the value of PbNLuc luminescence expressed in relation to the PbFLuc value. **c** Curve of serial dilutions of parasites (1–1000) at 12, 24, 36, 48 and 56 h of development in HepG2 cells. Six sets of 30,000 HepG2 cells were infected with 5000 sporozoites. Parasite numbers were calculated based on mCherry fluorescence, and total parasite load per well calculated. Cells were detached at each indicated time post-infection, and diluted to each indicated values. **d** Representative IVIS image of luminescence following the kinetics of infection at 12, 24, 36, 48 and 56 h post-infection following infection of HepG2 cells with an initial inoculum of 10,000 sporozoites. **e** Curve showing kinetics based on fluorescence measurements of total numbers, and sizes of at least 300 parasites at 6, 12, 24, 36, 48 and 56 h post-infection. **e**, **f** Represent the fluorescence-based quantification of (**d**). Parasite sizes (areas, μm^2^) were calculated based on acquired fluorescence images. **f** Curve showing kinetics of luminescence alone, corresponding to cells measured in (**e**) [luminescence measured in photons per second (p/s); ***p < 0.001; *error bars* in all graphs represent standard deviations (SD)]

Having proven the use and sensitivity of NanoLuc in mosquito stages, next liver stages of infection were assessed in vivo and in vitro. HepG2 cells seeded in 24 well-plates were infected with 10,000 PbNLuc sporozoites per well, and imaged by fluorescence microscopy at 6, 12, 24, 36, 48 and 56 h post-infection (Fig. 4a shows representative images and a schematic (adapted from [104]) of liver stage development). To exclude differences in luminescence due to differences in overall development or sizes, PbmCherry, PbFLuc and PbNLuc parasite sizes were measured at 6, 12, 24, 36, 48 and 56 h post-infection, and infection success and survival (i.e. total number of HepG2 cells infected at each time point) were quantified by fluorescence. No differences were detected between PbmCherry, PbFLuc and PbNLuc, neither in terms of size, nor successful liver stage completion (detached cell formation).

In order to compare luminescence intensities of PbFLuc and PbNLuc, HepG2 cells in 6 wells of a 24-well plate were infected with an equal number of sporozoites of either one of the strains. At 40 h post-infection these cells were lysed and their luminescence quantified. PbNLuc showed 140-fold higher luminescence than PbFLuc (Fig. 4b). Next, in order to determine the detection limit for liver stages, PbNLuc-infected HepG2 cells were detached, total parasite numbers estimated, and serial dilutions generated for each time post-infection, ranging from 1 to 1000 parasites for each time point (i.e., 6, 12, 24, 36, 48 and 56 h post-infection). The assay was successful in detection of single liver stage parasites at all times post-infection, and enabled differences (Fig. 4c) in the range of tens to hundreds to be detected. This level of sensitivity has not been reported for firefly luciferase, nor was it ascertained in the present work.

Next, kinetics of liver stage infection in vitro throughout pre-erythrocytic development in terms of growth and survival, were determined by luminescence (Fig. 4d). HepG2 cells were infected with an initial inoculum of 10,000 sporozoites per well. Fluorescence images of at least 100 infected cells per time point were acquired for size measurement. Ten wells of a 24 well-plate were assessed by fluorescence imaging for quantification of parasites for each time point (shown in Fig. 4e). Finally cells were lysed at 6, 12, 24, 36, 48 and 56 h post-infection, and bioluminescence measured (Fig. 4f). Each time point was repeated in 10 wells in three independent experiments. Total parasite sizes and numbers at 6 h post-infection were set as 100 % (Fig. 4e, f). While parasite numbers decreased significantly already at 12 h post-infection (50 % reduction), a dramatic decrease in numbers (of about 80 % compared to 6 h post-infection) was observed at 24 h post-infection. Parasite number remained relatively constant at from 24 to 36 and 48 h, and showed only a 2 % further decrease at 56 h post-infection, potentially explained by a small number of detached cells occurring at this relatively early egress time point (Fig. 4e, black line). Conversely, parasite sizes at 6 and 12 h post-infection were almost equal, while size increased 3-, 6-, and 10-fold at 24, 36 and 48 h respectively, compared to parasite sizes at 6 h post-infection. The parasite size increase between 48 and 56 h was only minimal (Fig. 4e, blue line). The overall result of these changes in parasite numbers and sizes, in addition to potential changes in promoter activity, resulted in variations in luminescence intensity through time, with total luminescence being lowest at 24 h post-infection, after which these values increased by 48 h to almost 300 % of the level measured at 6 h post-infection (Fig. 4f).

### Bioluminescence-based analysis of PbNLuc liver egress in vitro by assessment of detached cells and merozoite counts

**Fig. 5:**
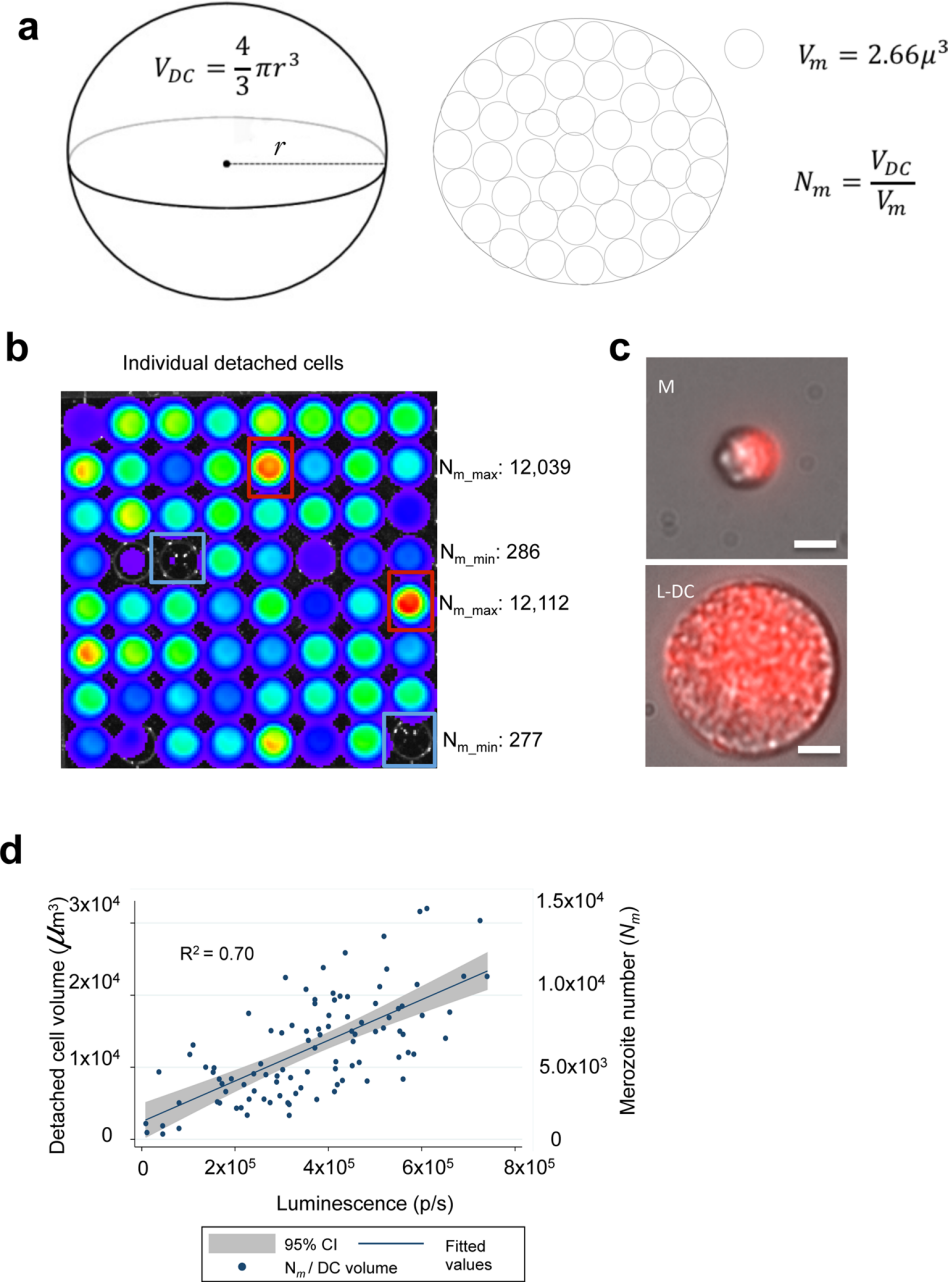
NanoLuc imaging of *P. berghei* detached cells allows calculation of merozoite numbers. **a** Schematic representation of a detached cell. The calculation of merozoite numbers within a detached cell is derived from measurement of the detached cell volume. **b** Representative bioluminescent image of 64 individual detached cells. **c** Fluorescence images of 2 types of detached cell accounting for variability in luminescent measurements. The top panel shows a merosome (M) with even distribution of merozoites within the cell body. The bottom panel shows a large detached cell (L-DC) with even distribution of merozoites within the cell body. Scale bar corresponds to 10 μm. **d** Scatter plot showing significant correlation of detached cell volumes and merozoite numbers (*y-axis*), with and PbNLuc luminescence (*x-axis*). 100 individual detached cells symbolizing successful completion of PbNLuc liver infections (represented by each *dot*) were detected by mCherry-based fluorescence microscopy, imaged, and then individually captured and lysed for luminescence imaging. The radius of the detached cell was measured, and the volume of the detached cell thereby estimated as described in (**a**) [luminescence measured in photons per second (p/s)]

While it was possible to investigate overall liver stage growth of PbNLuc parasites by bioluminescence, a marker of fully successful liver stage development is the successful production of merosomes and detached cells in vitro. Detached cell formation in vitro, like merosome formation and parasite egress from the liver in vivo, occurs upon formation of merozoites [101]. Therefore whether a PbNLuc-based luminescence is sensitive enough to allow estimation of numbers of merozoites in individual detached cells, was investigated next. While individual merozoites were not quantified, their number was estimated by measuring the detached cell volume as previously published [101], and as shown in Fig. 5a. Figure 5b shows representative bioluminescence images of individual detached cells of various sizes (and therefore, different numbers of merozoites). Each well has a single detached cell. While the median calculated number of merozoites in 100 detached cells was 4500 (with the average detached cell volume being 11,780 μm^3^), Fig. 5b shows clear outliers (marked) representing both larger detached cells or those with much higher merozoite numbers (marked in red outline), and also those extremely small or with very reduced merozoite numbers (marked in blue outline). Figure 5c shows representative fluorescence images of these extreme size variations between merosomes (M) and large detached cells (L-DC). Figure 5d shows the correlation of detached cell luminescence with the volume calculated by fluorescence imaging, and the number of merozoites calculated on the basis of the volume estimated.

### Liver egress and determination of pre-patency period

**Fig. 6:**
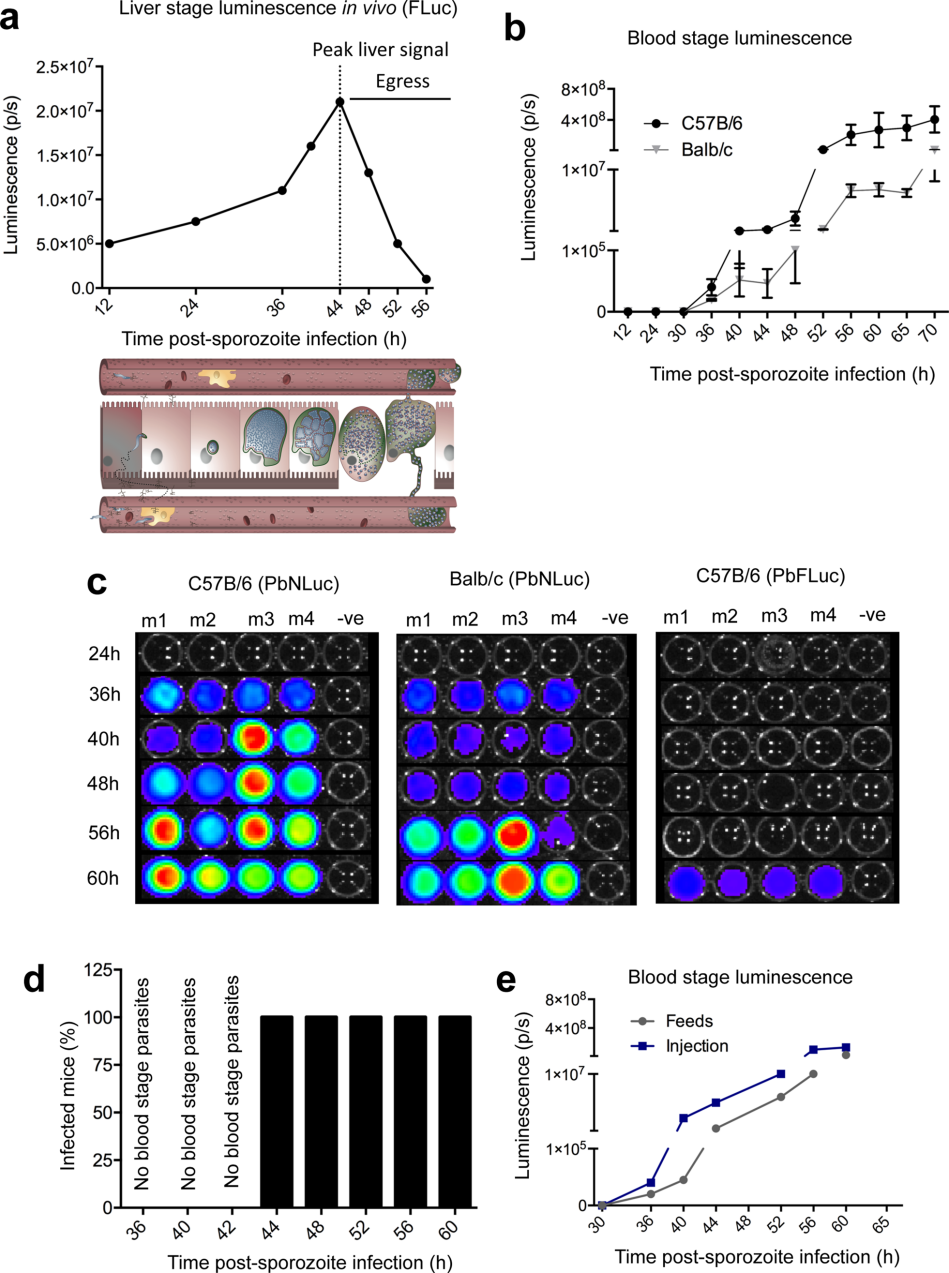
Bioluminescence imaging of *P. berghei* liver stage egress. **a** Luminescence values obtained from in vivo imaging of C57BL/6 mice infected with 5000 sporozoites of PbFLuc. Dotted line shows the peak of luminescence after which the first obvious decrease in signal from the mouse liver in vivo after around 48 h occurs. **b** Egress measured by first appearance of parasites in the periphery. 2 μl of blood were obtained via tail vein puncture and lysed in 20 μl of 1 × PLB for bioluminescence measurement. Luminescence values of peripheral blood from C57BL/6 and Balb/c mice infected with 5000 PbNLuc sporozoites obtained at the indicated time points post-sporozoite injection until 70 h, showing the kinetics of egress as measured by PbNLuc. **c** Representative luminescence-based assay of egress at the indicated time points in 4 separate C57BL/6 and Balb/c mice. 2 μl of blood were obtained via tail vein puncture and lysed in 20 μl of 1 × PLB for bioluminescence measurement. While egress of PbNLuc is detected earliest at 36 h post-infection, egress of PbFLuc is detected for the first time at 60 h post-infection. **d** Success of passages of liver-egressed parasites from 36 h onwards into naïve mice. Six C57BL/6 mice were infected with 5000 sporozoites each, and at 36, 40, 42 44, 52, 56 and 60 h post-sporozoite injection, 20 μl of blood removed, diluted in 200 μl of 1 × PBS and the 220 μl intravenously injected into 3 naïve mice. It was later (at 70–90 h post-infection) assessed whether the inoculum had produced an infection in these recipient mice. **e** Comparative kinetics of egress as measured by luminescence in the blood, in mice infected by intravenous injection of 5000 sporozoites, or by mosquito feeds (5 mosquitoes) [luminescence measured in photons per second (p/s); ***p < 0.001; *error bars* in all graphs represent standard deviations (SD)]

Given the high sensitivity of NanoLuc detection, liver stage egress in vivo was re-investigated. While the most sensitive assay reported to date states that the first hepatocyte-derived blood stage parasites can be detected earliest at 60 h post-sporozoite injection [15], previously reported in vivo luminescence assays [39], as well as in vivo measurements performed here using PbFLuc (Fig. 6a) suggested a significant decrease in liver loads from 42 h post-infection onwards in untreated mice. This suggests that egress has potentially already begun occurring at this time point. Whether the previously reported lack of detection of parasites in peripheral blood between 42 and 60 h post-sporozoite injection is due to very low parasite densities (and therefore undetectable by firefly luciferase), or due to migration of merosomes to specific tissues (away from the periphery), was not known. Following infection of C57BL/6 (permissive) and Balb/c (refractive) mice with 5000 PbNLuc sporozoites, luminescence in peripheral blood was measured at 12, 24, 30 h post-infection, and every 4 from 36 h post-infection onwards, until 70 h post-infection. Using the maximum sensitivity setup for NanoLuc, luminescence signal was detected from 36 h post-infection in some mice, and by 40 h post-infection and at all subsequent time points, in all mice. Consistent with the known permissiveness of C57BL/6 mice for liver stages, and the refractoriness associated with liver stage infections in Balb/c mice, the detectable levels of luminescence in peripheral blood were on average 100-fold lower in Balb/c mice, but nevertheless the earliest signal suggesting egress, occurred at 36 h in both mouse strains (Fig. 6b). In parallel to PbNLuc infections, a separate group of C57BL/6 mice was infected with PbFLuc. As published in previous work [15], firefly-luciferase parasites were only detected at 60 h post-infection, but not earlier (Fig. 6c). Dynamics of egress displayed by PbNLuc and PbFLuc are shown in Fig. 6c.

In order to determine whether parasites (merosomes, free merozoites or early infected RBCs) present in peripheral blood from 36 h onwards were infectious to naïve mice thereby confirming egress, 20 μl of blood were extracted from the sporozoite-infected C57BL/6 mice at 36, 40, 42, 44, 52, 56 and 60 h post-infection, and injected intravenously into three naive C57BL/6 mice for each time point. While blood extracted at 36, 40 and 42 h did not lead to blood stage infections in the naïve mice, blood extracted at 44, 52, 56 and 60 h resulted in infection in all three recipient mice (Fig. 6d). Careful analysis by fluorescence microscopy allowed observation of amorphous, relatively large structures (potentially merosomes) at 36 to 42 h post-infection, which nevertheless did not lead to infection in the recipient mice.

Next it was determined whether differences exist in egress times of PbNLuc parasites in mice injected with sporozoites by intravenous injection, or mice on which infected mosquitoes fed. No differences in egress times were observed, despite mice infected by intravenous injection of 5000 sporozoites displaying at least tenfold higher signal than mice infected by feeds with five mosquitoes (Fig. 6e).

### Bioluminescence-based analysis of PbNLuc blood stages, and limit of detection

**Fig. 7:**
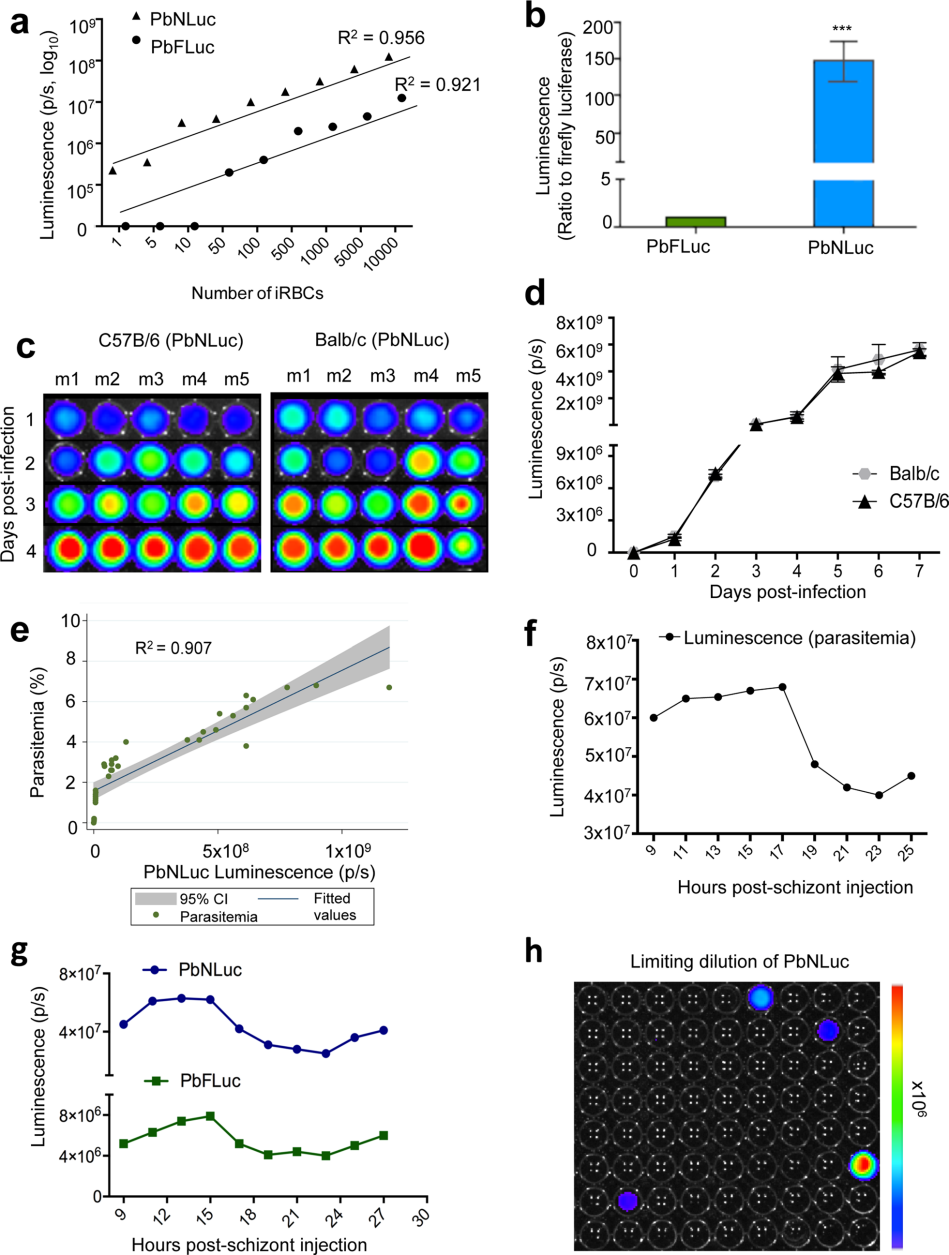
Bioluminescence imaging of *P. berghei* blood stages. **a** Luminescence values from various concentrations of iRBCs from PbNLuc- and PbFluc-infected mice, ranging from 1 to 10,000 iRBCs (values shown in logarithmic scale). **b** Ratio of luminescence of PbNLuc iRBCs compared to PbFLuc iRBCs showing a 150-fold higher signal. 1000 parasites were isolated from a drop of blood of synchronously infected mice at 18 h post-infection. The value for PbFLuc luminescence was set to a value of 1, and the value of PbNLuc luminescence expressed in relation to the PbFLuc value. **c**, **d** Parasitaemia curve as measured by bioluminescence, in Balb/c and C57BL/6 mice. 9 Balb/c and C57BL/6 mice were infected with 10^5^ parasites, and their parasitaemia followed daily. At every time post-infection, 20μl of blood were taken by tail vein puncture, and luminescence was assessed; **c** shows representative images of 5 mice followed in (**d**). **e** Correlation between parasitaemia and luminescence at various times post-infection and various parasite burdens. Following injection of 10^5^ parasites into mice, 25 μl of blood were obtained by tail vein puncture for measurement either by luminescence (*x-axis*) or Giemsa stain of thin blood smears from which parasitaemia was quantified using a light microscope (*y-axis*). **f** Bioluminescence signal from PbNLuc synchronous infections. 10^6^ schizonts were injected into C57BL/6 mice, and 20 μl of blood collected bi-hourly from 9 to 27 h post-infection, showing sequestration from 18 to 25 h post-infection. **g** Bioluminescence signal from blood of PbNLuc and PbFLuc co-infected mice, as measured bi-hourly from 9 to 27 h post-synchronous infections, showing marked schizont sequestration of both parasite lines from 18 to 25 h. **h** Graphical representation of a limiting dilution leading to isolation of single parasites. The graph shows 5.5 % positivity. [***p < 0.001; *error bars* in all graphs represent standard deviations (SD)]

As for other stages, the sensitivity of NanoLuc for low parasite densities was tested in blood stages of infection with PbNLuc. Limiting dilution ranging from 1 to 10,000 parasites showed that even single parasites can be detected, and that parasite number and luminescence are highly correlated (R_2_ = 0.956) (Fig. 7a). While infections with PbFLuc hold equally high correlation (R_2_ = 0.921), its signal intensity is at least 100-fold lower, and does not allow detection of single parasites (Fig. 7a). To determine differences in sensitivity of PbNLuc compared to PbFLuc in blood stage infections, a drop of blood was obtained by tail vein puncture, and 1000 parasites were isolated by dilution in 1xPBS. Figure 7b shows that PbNLuc has 150-fold higher signal than PbFLuc, supporting the results with the other parasite stages. To determine whether differences in parasite development exist in Balb/c and C57BL/6 mice, parasitaemias arising from infection with 10^6^ schizonts by tail-vein injection were followed by luminescence and Giemsa stains for 7 days. No significant differences concerning blood stage infection were found between the two mouse strains (Fig. 7c, d). Figure 7c shows representative images from 5 different mice at days 1–4 post-infection.

While assessment of various sequential parasite dilutions (Fig. 7a) showed high correlation with luminescence, whether in vivo parasitemia correlates with luminescence was further explored. Figure 7e shows the correlation of luminescence with parasitaemia calculations assessed by manual counting of Giemsa-stained thin blood smears. Parasitaemia and luminescence values showed a strong correlation (R^2^ = 0.907) (Fig. 7e).

The sensitivity of the NanoLuc assay allows determination and comparison of multiplication rates between mice and parasite strains. Previous work has explored the use of luminescence to detect schizont sequestration in synchronous infections in relation to the day light-cycle [16, 17]. In this work, blood stage parasites from a donor mouse were cultured in order to purify schizonts. Schizonts were injected into naïve mice to establish synchronous infections that were followed bi-hourly over 27 h, when specific times would correspond to synchronous ring, trophozoite, and schizont stages. Figure 7f shows results of these bi-hourly luminescence measurements of peripheral blood parasitaemia of mice infected with PbNLuc schizonts. The drop in parasitaemia from 18 h onwards, coinciding with the beginning of schizont sequestration in the vasculature, is clearly identified.

In order to test the use of PbNLuc in multiplex assays using two different luciferases, mice were co-infected with PbNLuc and PbFLuc. Figure 7g shows the comparative values of luminescence of both luciferases in the same mice, clearly showing sequestration of schizonts from both strains at around 18 h after synchronous infection.

Finally, to test further potential applications of the NanoLuc-expressing parasites, since even single PbNLuc parasites can be identified due to the strong luminescence intensity of NanoLuc, and limiting dilution is a routinely used method for clonal selection of genetically-modified parasites, it was determined whether identification of single parasites expressing NanoLuc, followed by their injection into naïve mice could lead to effective infection. By limiting dilution, the estimated volume containing 1 parasite was isolated and deposited into a 96-well plate. NanoLuc was diluted in 1 × PBS rather than lysis buffer to ensure the viability of the parasites, and 4 of the 96 (i.e. 5 %) wells were found to be positive (Fig. 7h). The full volume of all four wells was isolated and injected into recipient mice. All caused effective infections in the recipient animals, suggesting the usefulness of the method to avoid large animal usage in limiting dilution experiments.

## Discussion

*Plasmodium* luminescent lines have been widely used to study parasite biology in vivo and in vitro, for applications ranging from evaluation of the effect of anti-malarial compounds to characterization of parasites with mutations affecting one or various stages of the life cycle. While firefly luciferase has been successfully and widely used for in vivo and in vitro applications, the novel enzyme NanoLuc has been reported to produce 150-fold higher luminescence intensities than firefly luciferase [70], making it an attractive tool for highly sensitive characterization of all *P. berghei* stages in the context of mutations, drug-treatments and as yet understudied phenomena such as parasite egress from the host liver. In this work, NanoLuc was found to be highly sensitive for detection of parasite densities down to single sporozoites, single liver stages in vitro, and single infected erythrocytes, and highly accurate in determination of overall parasite loads at all infection stages. Such sensitivity and accuracy has not previously been reported with the widely used firefly luciferase. While various mutations in the *P. berghei* genome produce drastic changes in terms of development or growth rates, others produce only mild effects that cannot be assessed without extremely sensitive tools. The benefits and applications of the PbNLuc transgenic line for the characterization of anti-malarial drugs and mutant parasites is discussed for each stage below in the context of the findings presented in this work.

Malaria transmission is a major focus of research as a target for anti-malarial interventions. Evaluation of *Plasmodium* oocyst burden in the midguts of *Anopheles* mosquitoes is one of the most important methods for investigating transmission. Most reported assays rely on manual counting of fluorescent oocysts, which is slow and error-prone given the complex organization and fluorescent background noise in the mosquito midguts. This challenge limits the scalability and throughput of the assay in general. In 2012, Delves and Sinden presented a semi-automated method for counting fluorescent oocysts that significantly increases the scalability and reduces evaluation times [103]. In this work, a comparison of PbNLuc sensitivity both with manual counts and the semi-automated fluorescence-based method was undertaken. A medium correlation between both methods was found, with PbNLuc producing a varied range of light output for the same hypothetical number of oocysts calculated by the semi-automated fluorescence-based method. Two reasons might contribute to this discrepancy: the inevitable variation in productivity of oocysts, and on the other hand, the high sensitivity of the NanoGlo™ assay detecting parasites that are not easily detectable by fluorescence microscopy. Although NanoLuc assays are slightly limited in the accuracy of quantitatively determining oocyst numbers, the assay gives a clear idea of the overall burden in the mosquito midgut. Firefly luciferase-expressing *P. falciparum* parasites have been generated in the context of transmission-reducing assays (TRAs) or transmission-blocking assays, and showed that luciferase-based TRA estimates, consistently mirror those based on microscopy read-outs [50, 105]. Given the higher sensitivity of PbNLuc, it is envisaged that upon generation of a marker-free parasite line, this tool will enable much more accurate calculation of the transmission-reducing or -blocking effects of drugs and genetic mutations in *P. berghei* knock-outs. In the context of calculating infection prevalence among mosquito pools, NanoLuc showed greater sensitivity than observer-based determination of fluorescence in the midguts, in particular if oocyst burdens were low. The sensitivity of the assay further allowed assessment of changes in oocyst size, maturation, and assumingly, promoter activity, between days 7 and 9 post-infection, showing that interventions or mutations producing even slight effects and not abrogation, as well as effects in sporogonic development, can still be evaluated with this method.

While the correlation of oocyst counts with luminescence values is somewhat limited by confounders including oocyst sizes in the midguts, sporozoite quantification and correlation with luminescence is straightforward. PbNLuc allowed detection of between 1 and 10 sporozoites, with a sensitivity not achieved by the PbFLuc counterparts. Equally in the context of transmission-blocking research, the assay can accurately quantify even slight variations in sporozoite burden among a mosquito population. Preliminary analyses are being undertaken to characterize the effect of treatment of mice with atovaquone, chloroquine and sulfadiazine (prior to mosquito feeds) on PbNLuc oocyst and sporozoite formation as measured by luminescence. These and other additional data will be published separately.

Aside from its applicability in the context of transmission-blocking drugs, PbNLuc is a promising line for the careful characterization of events occurring in the gametocyte-ookinete-oocyst transition, as well as the kinetics of sporozoite development, egress, and migration to the mosquito salivary glands. So far, *Plasmodium* mutants have been generated to characterize the role of multiple proteins involved in mosquito-stage development. Examples of targeted proteins found to play a role in mosquito-stage *Plasmodium* development include, among others, those necessary for gamete release prior to the production of ookinetes; those necessary for ookinete formation and escape from the midgut and those involved in sporozoite egress, migration and motility. Characterization of the kinetics of *Plasmodium* mosquito stage development have so far largely relied on fluorescence-based microscopy, and only later on firefly bioluminescence imaging. The high bioluminescent signal of NanoLuc would enable much more sensitive evaluation of phenotypes of mutant *Plasmodium* parasites, potentially allowing identification of modifications leading to even relatively mild phenotypes. The phenotypes potentially measurable by PbNLuc include developmental arrest within the mosquito; deficient ookinete formation; deficient or abrogated oocyst formation; deficient sporozoite egress, and overall reductions in parasite loads at oocyst and sporozoite stages.

A further application where PbNLuc would be advantageous is in exploring the dynamics of *P. berghei* transmission from mosquitoes to mice at the skin interface. Transmission dynamics have previously been assessed by approaches such as quantification of sporozoites released by mosquitoes into liquid media or glass slides [106], or determination of sporozoite 18S rRNA [107], or beta-galactosidase [108] expressed in transgenic sporozoites following mosquito feeding on mouse skin. Since then, complex microscopic approaches have been developed, including the use of intravital and spinning disc microscopy [109]. While these provide accurate measurements of sporozoite dynamics, NanoLuc offers the possibility of assessing sporozoite localization in, and time of clearance from, the host skin, which would be advantageous in the context of host and parasite mutations affecting sporozoite motility, immune defence, or skin traversal. While limitations for the use of NanoLuc in in vivo imaging of deep organs will be discussed below, the use of this enzyme in the skin and respiratory tract has been reported for other organisms, with rather successful results [73, 78].

Following injection into the host skin, and upon reaching the host liver, each *Plasmodium* sporozoite is able to asexually replicate within host hepatocytes resulting in the production of thousands of erythrocyte-infectious merozoites. The liver stage, although clinically silent, is a bottleneck for parasite development, hence ideal for anti-malarial interventions. In the context of liver stage infections in vitro, NanoLuc showed great sensitivity, detecting as little as one parasite in all pre-erythrocytic developmental stages investigated. While fluorescence microscopy has been extremely valuable for the careful dissection of mechanisms altered upon mutations, or drug treatment affecting either the parasite or the host [8, 11, 15, 34–37, 39, 40, 51], luminescence imaging provides a quick and sensitive alternative to measuring overall effects of drugs and mutations on parasite development, host-pathogen interactions, parasite survival, and successful completion of the pre-erythrocytic stage. A relative limitation of this assay in vitro, is that it does not allow differentiation between parasite numbers and sizes, but rather gives only a value of luminescence which can be confounded by the effects of promoter activity. Nevertheless, the assay is highly sensitive in showing the overall kinetics of liver stage development from sporozoite injection to merosome formation. In the present work, it enabled visualization of major drops in luminescent signal early on in infection, corresponding to a decrease in parasite numbers (not sizes). This is consistent with the fact that a massive number of parasites are eliminated by host cell autophagy, prior to 24 h post-hepatocyte invasion [39]. Following this elimination, surviving parasites will be able to develop normally with little losses in the subsequent hours preceding liver stage egress.

Mutations or drugs affecting the parasite, including sporozoite invasion, sporozoite motility, liver stage growth, host immune mechanisms, establishment of a parasitophorous vacuole membrane (PVM), egress, or merozoite number per merosome, can be easily and quantitatively explored using NanoLuc. Additionally, the effect of drugs affecting the host during liver stage infections, such as those targeting host cell autophagy, can also be accurately evaluated with the highest sensitivity so far reported. Work to evaluate the effects of drugs such as rapamycin and chloroquine (an autophagy enhancer and inhibitor, respectively) in the context of *P. berghei* egress from the liver is presently ongoing. While point-by-point characterization of parasite counts might still require parallel assessment with microscopy, NanoLuc provides a considerable advantage over microscopy for high-throughput screening.

Although the liver stage in general is an attractive target for blocking parasite development, liver stage egress is the key interface between the asymptomatic pre-erythrocytic stage and the clinically symptomatic blood stages. Although our group pioneered research on *Plasmodium* egress from host livers a decade ago [101], to this date relatively little is known about host and parasite mechanisms affecting egress. The so-called pre-patent period is defined as the time between sporozoite inoculation and the appearance of *Plasmodium* parasites in the blood. So far, the pre-patent period has been assessed by time-consuming or subjective techniques including PCR, fluorescence, and light microscopy. In 2014, Zuzarte-Luis et al. published a luminescence assay based on firefly luciferase whereby first blood stage parasites were detected at 60 h post-sporozoite inoculation into mice [15]. Interestingly, careful characterization of development and egress in previous in vivo work using firefly luciferase detection by IVIS, showed that maximal luminescent signal in the liver is detected at 44 h post-infection, followed by a gradual decrease in luminescence from 46 h onwards [39]. The sharpest decrease in luminescence in vivo occurs between 48 and 56 h post-infection. Previous work by Sturm et al. confirmed egress by intravital microscopy, as early as 42 h post-infection. Even if the circulating parasite ‘forms’ in the peripheral bloodstream are merosomes *en route* to sites such as the lungs as has been previously proposed [110], it is indeed intriguing that a gap of at least 16 h seems to exist between detection of a decrease in liver luminescence in vivo, and detection of erythrocytic stages in the bloodstream.

Following inoculation of mice with PbNLuc sporozoites, upon monitoring the peripheral blood for detection of egressed parasites, luminescence signals were detected as early as 36 h post- sporozoite inoculation, and consistently strong signals at 40 and 42 h post-infection supporting previous observations of egress done by intravital imaging (Heussler et al, unpublished). As in this study mice were infected in parallel with PbFLuc, previously reported observations of an absence of signal in peripheral blood at all times prior to 60 h post-sporozoite infection were confirmed [15]. This discrepancy, together with the assessment of the enhanced threshold of parasite detection of NanoLuc, suggests that lack of detection of parasites in the peripheral blood using firefly luciferase at time points prior to 60 h post-sporozoite inoculation is due to insufficient assay sensitivity. To confirm the infectious nature of PbNLuc egressed from the liver, peripheral blood from sporozoite-infected mice was isolated starting at 36 h post-infection. From 44 h post-infection onwards, inoculation of this blood into naïve mice resulted in the successful establishment of blood stage infections thereby confirming the presence of parasites since these early times (prior to 60 h as previously believed). On the other hand, inoculation of naïve mice with the blood obtained at 36–42 h post-sporozoite injection in the donor mice did not result in positive infections. It is tempting to speculate that the luminescent signal in peripheral blood of the donor mice at 36–42 h post-sporozoite injection is arising from newly formed merosomes that either contained non-infectious, immature merozoites or that, upon injection of these merosomes into a naïve mouse, the merosomes are eliminated for example in the spleen.

Interestingly, upon studying liver stage egress rates in 4-hourly periods by monitoring peripheral blood, a significant peak in signal increase in peripheral blood occurring at 56 h post-sporozoite injection was observed, coinciding with the sharpest decrease in liver stage signal as described previously in vivo [39]. This suggests that initially, egress occurs at very low rates until 56 hpi when either large-scale egress of parasites from the liver occurs, or merosomes transported to other tissues, such as the lungs, rupture at this time point, and release large numbers of merozoites into the bloodstream. This sharp increase would explain the high signal detected in the blood at 60 h post-sporozoite injection, also detectable by firefly luciferase. The PbNLuc parasites, and their 150-fold higher signal than PbFLuc, appear to be an extremely valuable tool in the context of evaluating the dynamics of egress upon parasite and host alterations, as well as parasite mutations affecting egress.

A further application explored in this work, was measurement of individual detached cells in vitro. Total quantification of detached cells is a measure of successful parasite development throughout the pre-erythrocytic stage. Analysis of drugs or mutations affecting parameters in parasites or hosts with downstream effects on detached cell-formation and egress in vitro so far relies on quantification of total detached cells by fluorescence microscopy, which is time-consuming and not very accurate. In addition, in this work NanoLuc was shown to be sensitive enough to detect signals from single detached cells, and to accurately differentiate between cells with different volumes and different loads of merozoites.

Finally, PbNLuc assessment in blood stages was equally sensitive as in other stages, allowing visualization of luminescent signal from single parasites, and an almost perfect correlation of luminescence intensity with peripheral blood parasitaemia. PbNLuc was tested in the context of sequestration of highly synchronized parasites, where bi-hourly measurements of peripheral blood showed marked decrease in signal from 18 to 25 h, corresponding with schizont stages in synchronous infections. Schizonts are able to sequester in the vasculature and are therefore absent from peripheral blood.

As proof of principle of multi-plexing for the PbNLuc line, measurements of sequestration of PbNLuc co-injected with PbFLuc were performed. The spectral profile of NanoLuc has an emission maximum at 460 nm, which is 100 nm blue-shifted with respect to Firefly luciferase. This is an advantage in terms of multiplexing, as both luciferases can be measured using different filters. Furthermore, each of the enzymes requires a different substrate, namely furimazine for NanoLuc, and luciferin for firefly luciferase. For *Plasmodium* research, a potential application of NanoLuc in the context of multi-plexing, aside of co-infections, is the generation of lines whereby each luciferase is expressed under promoters specific to different parasite stages and sub-stages in terms of kinetics and localization. So far, staging is reliant on subjective, expensive, or extremely time-consuming techniques, and therefore this would be a largely beneficial tool of research.

The implementation of blood stage anti-malarial drug screening by luminescence has been widespread. Due to the higher sensitivity of detection of PbNLuc compared to PbFLuc, more sensitive assessment of anti-malarial compounds can be performed using this newly introduced line. The kinetics of clearance potential for various anti-malarials at various parasite stages is currently being explored using PbNLuc.

Finally, a further avenue for the application of this parasite line explored in this present study, is its use for limiting dilution assays for *P. berghei* sub-cloning. At present, a major hindrance for sub-cloning by limiting dilution of blood stages is that large amounts of mice are required for injection, theoretically of a single infected iRBC which will give rise to a clonal population. Since the use of PbNLuc parasites allow visualization of single iRBCs without the need for lysis, it was possible to perform limiting dilutions in 96-well plates and assess luminescence to detect the presence or absence of parasites prior to injection, thereby heavily reducing the mouse demand.

Overall, the advantages of NanoLuc in vitro surpass other luminescence and fluorescence assays for scalability, sensitivity and rapid quantitation. Furthermore, NanoLuc exists in a secreted and non-secreted form. The secreted form has been used for *P. falciparum* in the context of studying the *Plasmodium* exportome and trafficking pathways [97]. PbNLuc parasites or other mouse *Plasmodium* strains expressing the secreted form of NanoLuc could potentially be used for similar studies of protein export, throughout all infection stages, including mosquito and liver stages. An additional application of NanoLuc in other fields has been in the context of BRET (Bioluminescence Resonance Energy Transfer) to study protein–protein and/or receptor-ligand interactions, which would be an extremely valuable tool in *Plasmodium* research. BRET was originally established using *Renilla* luciferase [111–117], however the large size and relatively lower brightness of *Renilla* in comparison to NanoLuc, have made the latter a powerful molecule, and the current molecule of choice for BRET. Aside from receptor-ligand interactions alone, other studies have used NanoLuc to study ligand-induced receptor internalization and downstream signalling. Other ‘binding’ processes in biology can be equally studied with NanoLuc, including antigen–antibody binding, lectin-carbohydrate binding, protein–protein interactions, and protein-nucleic acid interactions, among others. In the context of *Plasmodium* research at the host and parasite interfaces, the use of BRET could be a powerful tool to replace other time-consuming or expensive assays currently in use.

Although the spectral profile difference of NanoLuc compared to the other luciferases is an advantage in vitro, the use of blue-shifted luciferases in vivo poses an important challenge, as short wavelengths do not readily penetrate mammalian tissues [70]. Although other work has used NanoLuc for characterizing events at skin level, imaging of deep organs in living mice, including the liver, lungs, spleen, and brain was attempted in this study and was found to be unsuccessful. The use of an equally bright assay in vivo would be highly advantageous for *Plasmodium* research to study low-density infections, and parasite migration within different organs of the mice. Previous work using targeted mutagenesis has led to the generation of red-shifted *Renilla* [118], Click beetle [47], and firefly [119] luciferases to increase their sensitivity upon in vivo use for imaging deep tissues. As red-shifting of these luciferases has proven to be successful, a similar approach might be envisaged for NanoLuc.

## Conclusion

Overall, PbNLuc is presented here as a parasite line with on average, 150-fold higher luminescence intensity than firefly luciferase. This novel tool enabled extremely sensitive and efficient evaluation of parasites at low densities, and detection of even small variations in parasite development at all stages. As such, this line represents a valuable tool for characterization of genetic mutations affecting various developmental stages throughout the entire *P. berghei* life cycle, as well as interventions such as testing of drug or vaccine targets, affecting parasite-host interactions, or drugs affecting host responses that hinder or enhance parasite development and growth.

## Abbreviations

BRET: bioluminescence resonance energy transfer
CCD: charged couple device
IVIS: in vivo imaging system
iRBC: infected red blood cell
LAB: luciferase assay buffer
L-DC: large detached cell
PLB: passive lysis buffer
RBC: red blood cell
S-DC: small detached cell
TEM: transmission electron microscopy
